# The benefits of carbon black, gold and magnetic nanomaterials for point-of-harvest electrochemical quantification of domoic acid

**DOI:** 10.1007/s00604-020-4150-x

**Published:** 2020-02-12

**Authors:** Joost L.D. Nelis, Davide Migliorelli, Safiye Jafari, Silvia Generelli, Javier Lou-Franco, J. Pablo Salvador, M. Pilar Marco, Cuong Cao, Christopher T. Elliott, Katrina Campbell

**Affiliations:** 1 Institute for Global Food Security, School of Biological Sciences, Queen’s University, 19 Chlorine Gardens, Belfast, BT9 5DL UK; 2 CSEM SA, Bahnhofstrasse 1, 7302 Landquart, Switzerland; 3 Nanobiotechnology for Diagnostics (Nb4D), Institute for Advanced Chemistry of Catalonia (IQAC) of the Spanish Council for Scientific Research (CSIC), Jordi Girona 18-26, 08034 Barcelona, Spain; 4 CIBER de Bioingeniería, Biomateriales y Nanomedicina (CIBER-BBN), Jordi Girona 18-26, 08034 Barcelona, Spain

**Keywords:** Marine toxin, Biosensor, Shellfish, Point of site, Point of care, Toxin, Food analyses, Amperometry, Material characterization

## Abstract

Gold nanostars (GNST), gold nanospheres (GNP) and carbon black (CB) are chosen as alternative nanomaterials to modify carbon screen-printed electrodes (c-SPEs). The resulting three kinds of modified c-SPEs (GNP-SPE, CB-SPE and GNSP-SPE) were electrochemically and microscopically characterized and compared with standardized c-SPEs after pretreatment with phosphate buffer by pre-anodization (pre-SPE). The results show outstanding electrochemical performance of the carbon black-modified SPEs which show low transient current, low capacitance and good porosity. A competitive chronoamperometric immunoassay for the shellfish toxin domoic acid (DA) is described. The performances of the CB-SPE, GNP-SPE and pre-SPE were compared. Hapten-functionalized magnetic beads were used to avoid individual c-SPE functionalization with antibody while enhancing the signal by creating optimum surface proximity for electron transfer reactions. This comparison shows that the CB-SPE biosensor operated best at a potential near − 50 mV (vs. Ag/AgCl) and enables DA to be determined with a detection limit that is tenfold lower compared to pre-SPE (4 vs. 0.4 ng mL^−1^). These results show very good agreement with HPLC data when analysing contaminated scallops, and the LOD is 0.7 mg DA kg^−1^ of shellfish.

**Electronic supplementary material:**

The online version of this article (10.1007/s00604-020-4150-x) contains supplementary material, which is available to authorized users.

**Graphical abstract:**
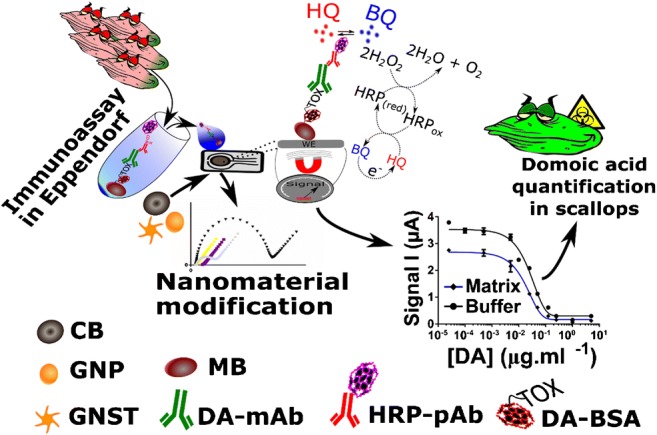
Schematic representation of the magnetic bead-based immunoassay for the quantification of domoic acid (DA) in shellfish with nanomaterial-modified screen-printed electrodes. CB, carbon black; GNP, gold nanospheres; GNST, gold nanostars; MB, magnetic beads; DA-mAb, anti-DA monoclonal mouse antibody; HRP-pAb, horseradish conjugated polyclonal goat anti-mouse antibody; DA-BSA, bovine serum albumin conjugated DA; HQ, hydroquinone; BQ, benzoquinone.

## Introduction

**Fig. 1:**
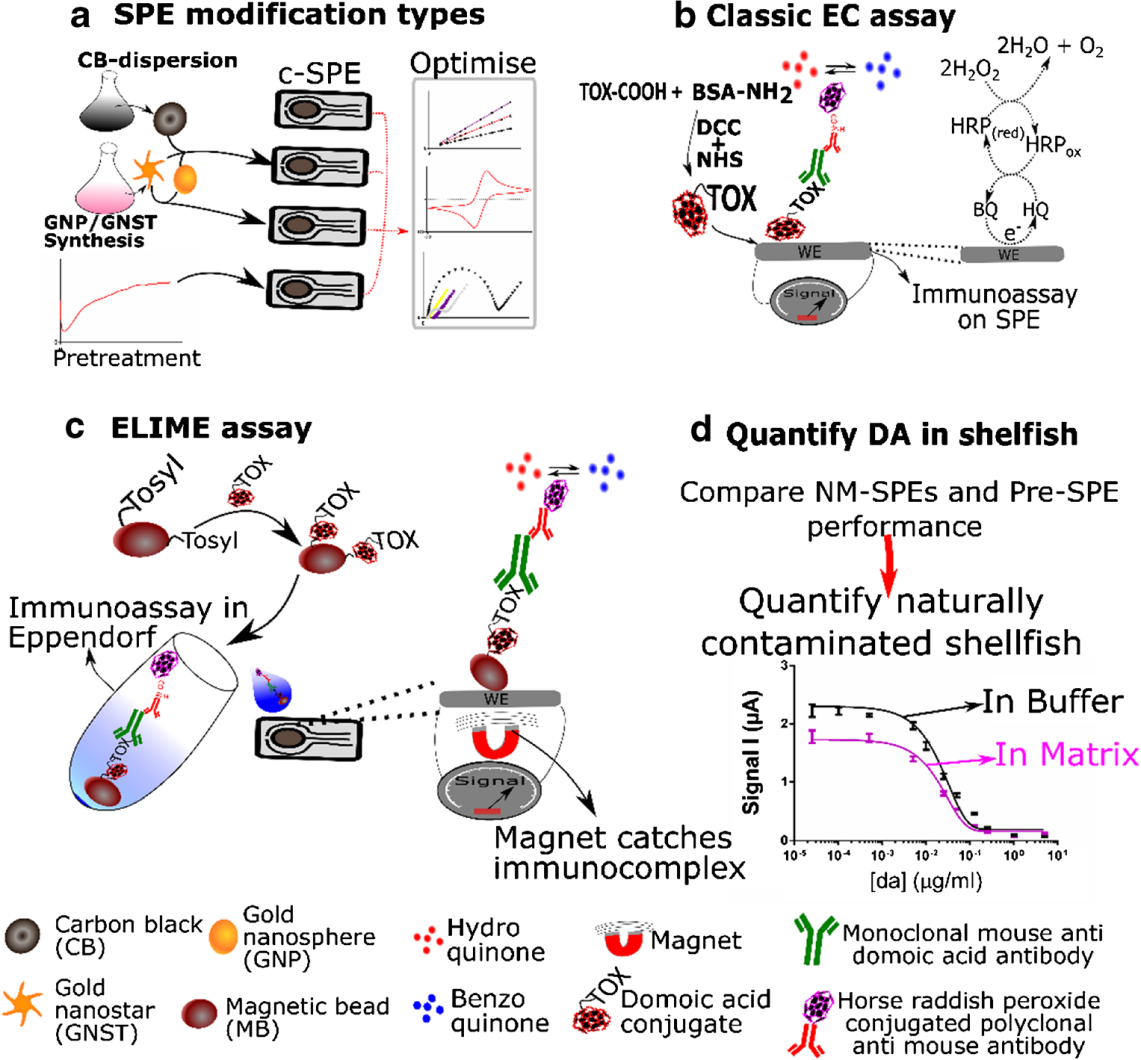
**a** Simplified scheme showing nanomaterial-modified screen-printed electrodes (NM-SPE) and pretreated SPEs (pre-SPE) optimization. **b** Scheme illustrating hapten synthesis and a direct immunoassay on pre-SPEs. **c** Workflow for magnetic bead (MB)/hapten synthesis and ELIME assay. **d** Synopsis of the final goal of this work

The use of nanomaterials to sensitize electrochemical methods has gained great interest in agricultural, medical and environmental sectors for in situ detection [1]. Nanomaterial-enhanced electrochemical sensing using graphene, graphene oxide or carbon nanotubes has been identified as the most sensitive detection system, surpassing mass spectrometry in terms of sensitivity by orders of magnitude. This is highlighted in a published report which compares over 900 optical, mechanical and electrochemical (bio)sensors [2]. Interestingly, no electrochemical sensors utilizing carbon black (CB) were reported in that database [2] or in an additional review compiling another 200 plus sensors on food contaminant detection [3]. Actually, only very limited use of CB in any electrochemical biosensor in any field was identified in an additional Scopus database search with 47 hits for the search term (“electrochemical” AND “biosensor” AND “carbon black”) against 3505 hits for the search (“electrochemical” AND “biosensor” AND “graphene” OR “carbon nanotubes”). Most of these CB-based electrochemical sensors are enzyme based while CB-SPE-based electrochemical sensors using a more general, antibody-based approach such as the work reported in [4] are less common. However, the material may be interesting for such sensors to lower working potential and increase sensitivity. Indeed, when combined with molybdenum disulfide, CB has been shown to greatly increase the sensitivity of a SPE-based sensor for the determination of o-diphenols in olive oil and considerably lower the working potential [5]. In another study, CB was used as electrochemical sensing material in a microchip for the sensitive amperometric quantification of phenol-derivated carbamate pesticides [6]. CB was also reported to outperform CNT, graphene oxide and reduced graphene oxide-modified SPEs in terms of electron transfer constant, redox reversibility, background currents [7] and cost-effectiveness: ~ 1 € kg^−1^ for CB [7] versus ~ 100–1000 € kg^−1^for graphene depending quality [8] and ~ 1000 € Kg^−1^ for CNT [9]). Thus, development of an exemplary antibody-based CB-SPE sensor is warranted. However, optimization of drop-casting CB onto SPEs (3 casts of 2 μL of 1 mg mL^−1^ CB dispersion [10]) allows room for improvement since multiple casting steps make the modification time-consuming and may increase capacitive current. In addition, detailed electrochemical and electron-microscopic characterization and comparison of CB-modified SPEs with SPEs modified with more classic nanomaterials, e.g. gold nanopheres (GNPs), with similar advantageous electro-catalytic properties [11], has, to the best of our knowledge, not been performed. Equally, reports on using gold nanostars (GNSTs) for SPE modification have not been identified. Yet, GNSTs may increase the active surface area, electro-catalytic activity and conductance similarly to GNPs [12]. GNST modification may equally allow optimum functionalization with recognition elements due to their enhanced surface/volume ratios [12]. Finally, it was noted that there is a gap between reported scientific literature and commercial development, whereby no commercially available electrochemical sensors capable of detecting major food contaminants exist [2]. This is most likely due to limited sensor benchmarking [2]. Hence, this work focused on comparing electrochemical performance through detailed electrochemical and microscopic analyses of SPEs modified with these novel nanomaterials with a recognized approach (e.g. anodic pretreatment [13]) to investigate the usefulness of these price-competitive alternatives to carbon nanotubes and graphene for c-SPE modification. Additionally, effort was invested in developing and validating a model biosensor for antibody-based determination of a food safety-related compound in its complex matrix with these nanomaterial-SPEs. To this end, performance of all nanomaterial-modified SPEs was compared (Fig. 1a). The marine shellfish toxin domoic acid was selected as a model low-molecular-weight compound with societal need for point-of-harvest determination. This societal need is due to the potential danger for food safety [14] and increased marine toxin occurrence caused by anthropogenic-induced eutrophication [15], climate change [16] and commercial shipping [16]. Sensitive electrochemical biosensors have previously been developed for DA (ng mL^−1^ range) using a classical approach (Fig. 1b) [17–19]. However, in these studies, no quantification of DA in naturally contaminated shellfish was reported. Direct immobilization of toxin conjugate to the working electrode (WE) of each individual SPE was used. This negatively affects sensor preparation time and storage space requirements. Furthermore, effective elimination of matrix can be challenging in such a setup due to the limited possibility for effective washing directly on the SPE. This may explain why use of washing buffer with exceptionally high amounts of surfactant (up to 5% tween-20) was reported [19]. To overcome this obstacle, the enzyme-linked immunomagnetic electrochemical (ELIME) assay [20] was optimized for DA determination (Fig. 1c). Advantageously, ELIME can be performed in vials using hapten-functionalized magnetic beads (MBs) (which are known to be stable at 4 °C for at least 2 months [5]). This system makes individual SPE functionalization with biological components superfluous. Another advantage of MBs is that they greatly facilitate isolation of analytes from a complex matrix through extensive washing of the MBs with the aid of a magnetic field. They also cause further signal enhancement through increased electron transfer due to better surface proximity of the analytes [21]. Finally, the performance of the two optimal nanomaterial-SPEs and pre-SPE was critically compared using ELIME. The most promising modified SPE was then used to quantify DA in naturally contaminated scallop samples (Fig. 1d). For these assays, a monoclonal anti-domoic acid antibody was used. This antibody showed no cross-reactivity towards naturally co-occurring toxins and compounds similar to DA that co-occur in shellfish in previous experiments [22, 23]. This being said, to determine the selectivity of the biosensor, possible interference caused by various marine toxins and DA similar compounds was equally tested here.

## Materials and methods

### Apparatus, electrodes, chemicals and software

Used reagents, apparatus and software are detailed in the supplementary material.

### Magnetic bead coating and bioconjugate preparation

The protocol for magnetic bead coating was adapted from Thermofisher™ and is detailed in the supplementary material 2.2.1*.* Bioconjugate preparation and hapten density determination was done following a procedure adapted from [24]*.* Briefly, conjugation was performed using *N*,*N*′-dicyclohexylcarbodiimide and *N*-hydroxysuccinimide bioconjugation while matrix-assisted laser desorption/ionization mass spectrometry (MALDI-MS) allowed to calculate the toxin density on the BSA conjugate which was found to be ~ 16 DA molecules per BSA molecule (Fig. S1). Further details of the protocol can be found in the supplementary information 2.2.2.

### c-SPE production, modification and CB dispersions

For c-SPE production, the reference electrode was printed with silver/silver chloride ink while the WE and counter-electrode were printed with graphite ink. Polymeric dielectric ink was used to insulate the electrodes and confine the WE. Ink modification with GNPs and GNSTs is detailed in supplementary material 2.3.1. For GNP/GNST, SPE modification by drop-casting 1.4 nM and 56 nM GNST or GNP solutions was mixed with DMF 1:1 and 5 μL was dropped on the WE. For SPE modification with CB various 1 and 2 mg mL^−1^, CB dispersions with DMF were made as detailed in supplementary material 2.3.2.

### Electrochemical characterization

Cyclic voltammetry (CV) experiments were performed in 0.1 M KCl in absence or presence of 5 mM ferro-ferricyanide [Fe(CN)_6_]^3−/4−^. Potential was scanned from − 0.3 to 0.6 V with a scan rate of 0.05 V s^−1^ and a step potential of 0.01 for 2 cycles except for active surface area determinations where scan rates were varied (0.01, 0.02, 0.05, 0.075, 0.1, 0.2, 0.3, 0.4 and 0.5 V s^−1^). Chronoamperometry experiments were performed at a fixed chosen potential for 30 s with 0.1-s time interval using different concentrations of hydroquinone (HQ). SPEs pretreatment was performed applying either + 1.5 or + 1.7 V to the SPE using 60 μL of PB-KCl from 30 to 180 s. For EIS experiments, 0 V applied potential versus V_OCP_, 100 kHz to 0.1 Hz frequency range and 10 mV amplitude was applied in 5 mM [Fe(CN)_6_]^3−/4−^ with 0.1 M KCl. Nyquist plots were fitted in the theoretical equivalent of Randles Circuit.

### ELIME assay

Various amounts of MBs (between 15 and 3.75 μg of MB per chronoamperometric measurement) coated with BSA or BSA-DA (at 40 μg of protein per mg of MB) in 200 mL PBS-Wash were placed in a magnetic rack (2 min) and supernatant removed. MBs were incubated with 200 μL reagent buffer (PBS with 1% BSA) containing concentrations between 0.0375 and 1 μg mL^−1^ of DA-mAb (1 h, RT, slow rotation). Next, the supernatant was removed using the magnetic rack (2 min). MBs were suspended in 500 μL PBS-Wash and placed in the rack after which the supernatant was removed. This wash step was performed 3 times and followed by incubation with HRP-pAb (varying between 1 and 7.8 μg mL^−1^) in 100 μL reagent buffer (30 min, RT, slow rotation) after which triple washing steps were repeated. Finally, MBs were suspended in 40 μL citrate buffer (0.05 M; pH 5). 10 μL of MB suspension (thus 3.75 up to 15 μg of coated MB per test depending on initial amount of MB used) was dropped on the WE of an SPE connected to the potentiostat onto which a magnet was clamped. Immediately after that 40 μL of citrate buffer containing 0.1 M KCl, 1 mM HQ and 1 mM H_2_O_2_ was carefully added and mixed covering all electrodes. After 2-min incubation chronoamperometry measurements were initiated (*n* = 3).

### Domoic acid extraction procedure

Homogenized scallop (1 g) free from DA as determined by HPLC analyses [22] was weighed out and mixed with 19 mL of MQ, shaken vigorously (5 min), then filtered using a 5-μM filter. The filtrate (20 μL) was added to 480 μL reagent buffer spiked with various concentrations of DA. DA concentration in naturally contaminated scallop samples was determined by following an identical extraction/analysis procedure.

### Nanoparticle synthesis, electron microscopy and ELISA assays

GNP synthesis followed the Turkevich method [25] while GNST synthesis was adapted from [12]. For the indirect competitive ELISA, a previously described protocol [26] was optimized and applied. For top morphology analyses, the SPEs were mounted on aluminium discs using copper tape. The mounted SPEs were imaged with a field-emission gun scanning electron microscope (FEG-SEM) at low energy (3 kV) and small currents to prevent charging effects. For transmission electron microscopy analysis, samples were placed on a Formvar carbon mesh (Agar Scientific). One hundred twenty kilovolts was used and various magnifications applied to visualize the GNST and GNP particles. Detailed descriptions of these protocols and electron microscope specifications are given in supplementary material 2.7.

### Selectivity

Selectivity of the biosensor for DA against the co-occurring marine toxins saxitoxin (STX), neosaxitoxin (NEO), gonyautoxin-2 (GTX-2), okadaic acid (OA) and tetrodotoxin (TTX) was tested. Additionally, the compounds l-glutamic acid (GA), l-glutamine (GluNH2), aspartic acid (Asp.A) and l-ascorbic acid (L-AA), which are structurally similar to DA and are usually present in shellfish tissue [22, 23, 27], was tested for their interference as well. A detailed description of the method used can be found in the supplementary material section 2.8.

## Results and discussion

### SPE modification with gold nanoparticles

**Fig. 2:**
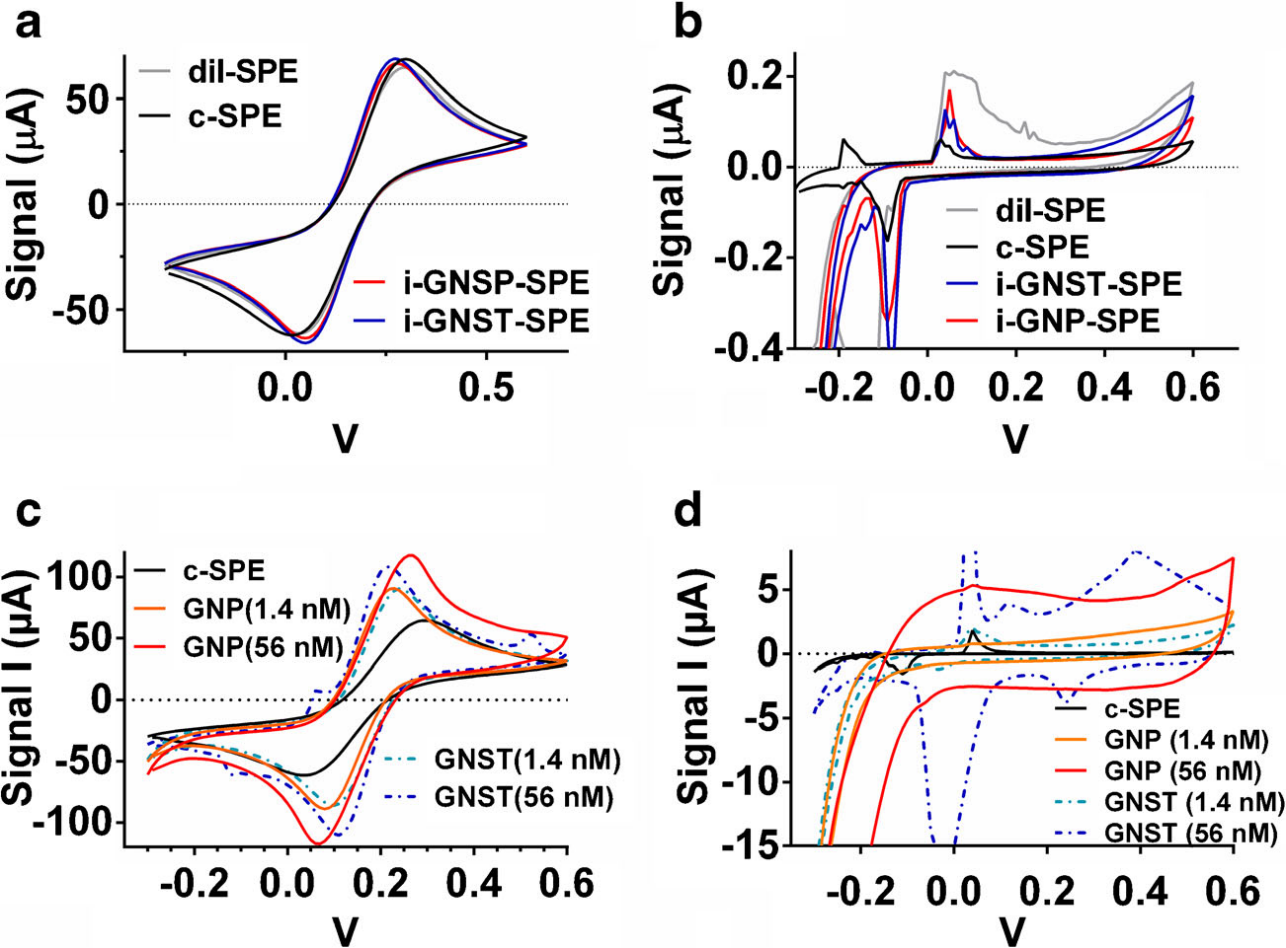
**a** Cyclic voltammogram (CV) in [Fe(CN)_6_]^3−/4−^ of SPEs printed with GNP/GNSTs mixed in the ink (concentration of particles used was 80 nM). **b** CV of same SPEs in phosphate buffer with KCl (PB-KCl). Black lines for standard graphite SPE (c-SPE), red for ink modified with GNP (i-GNP-SPE), blue for ink modified with GNST (i-GNST-SPE) and grey for a negative control using water instead of nanoparticle solution for ink modification (dil-SPE). **c** CVs in [Fe(CN)_6_]^3−/4−^ of SPEs modified by drop-casting with GNSTs (broken lines; GNST-SPE) and GNPs (full lines; GNP-SPEs). Five microliters of two concentrations for each gold nanoparticle type was drop-casted on the SPE. Namely 1.4 nM GNP (orange); 1.4 nM GNST (light blue); 56 nM GNPs (red) or 56 nM GNST (dark blue) colloid nanoparticle solutions. **d** CVs of these GNP-SPEs and GNST-SPEs in PB-KCl. **a**–**d** Voltage sweep was between − 0.3 and 0.6 V. Step rate was 50 mV s^−1^

Successful synthesis of GNPs was confirmed using UV-VIS and TEM analyses (Fig. S2). A detailed analysis can be found in the supplementary material section 3.1. CV was performed to characterize the electro-catalytic activity and reversibility for c-SPE and the SPEs with ink modified with GNSTs or GNPs (i-GNST-SPE and i-GNP-SPE) at 2 concentrations (2 and 80 nM). Only a slight increase in electrochemical reversibility and catalytic activity towards the redox couple [Fe(CN)_6_]^3−/4−^ was observed at the highest concentration (Fig. 2a). Furthermore, all ink modifications caused higher background noise, which may increase background noise for the BQ detection in the immunoassay (Fig. 2b). Thus, bulk modification of c-SPEs with gold particles was not pursued further. CV analysis of GNP/GNST-modified SPEs by drop-casting instead of mixing in the ink did however show improved electrochemical activity and reversibility in function of the added particle concentration (Fig. 2c). Capacitive currents equally increased at highest GNP and GNST concentrations compared with c-SPE (approx. 5 μA). However, that increase was limited compared with the absolute amount of signal increase observed (approx. 50 μA) (Fig. 2d).

### Optimization of SPE modification and pretreatment procedure

Further detailed EIS, CV, SEM characterization and active surface calculations (based on the Randle-Sevcik equation for reversible redox processes) were performed to optimize the modification of the SPEs. Respective text and figures on these optimizations are given in the supporting material (section 3.1; Fig. S3-S8). In short, the following experimental conditions were found to give the best results and were used from hereon: dropping 5 μL of the 56 nM GNP solution on the WE for gold nanoparticle modification. Dropping 5 μL of a 2 mg mL^−1^ CB dispersion on the WE for CB-SPEs. Applying a 30-s SPE anodic pretreatment at 1.7 V for pre-SPEs. GNSTs tended to aggregate and caused sub-optimal performance. Thus, GNST modification was no longer used.

### EIS analyses of nanomaterial-modified SPEs and pretreated SPE

**Fig. 3:**
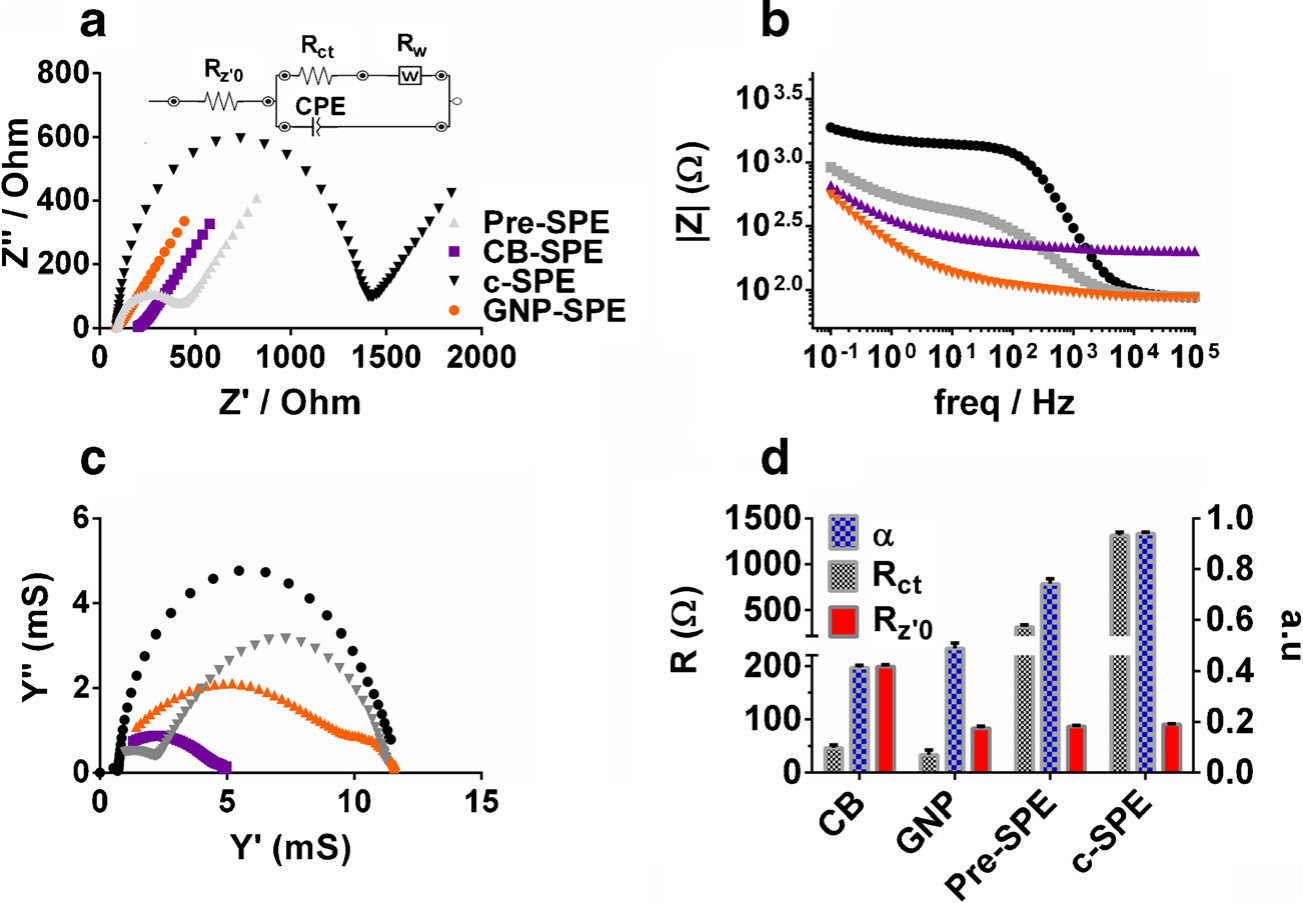
**a** Nyquist plot of the EIS analysis at the CB-SPE, GNP-SPE, pre-SPE and c-SPE using [Fe(CN)_6_]^3−/4−^ in 0.1 MKCl. The model used for fitting is shown inset. Frequency decreases from left to right. **b** Bode plot of the same data visualizing absolute impedance against frequency. **c** Complex admittance plot of the same data of imaginary admittance (Y″) against real admittance (Y′). **d** Bar chart showing the resistance of Rct Rz′0 and α exponent values of the CPE calculated from data fitting using the model as shown in **a** (*n* = 3)

Electric parameters of pre-SPEs are compared with optimized GNP-SPE, CB-SPE and c-SPE using EIS analyses (Fig. 3a–d). The Nyquist plots (Fig. 3a) were fitted against an adapted Randles circuit (capacitor was replaced by a constant phase element (CPE) (Fig. 3a inset). In this model *R*_z′0_ is the resistance where the Nyquist plot curve intercepts the real impedance axes. *R*_z′0_ represents a combination of electrolyte, intrinsic material and contact resistance [28]. *R*_z′0_ can equally be seen in the bode plot (Fig. 3b) as the asymptote in the high-frequency range where impedance of the CPE borders zero. Charge transfer resistance (*R*_ct_) is represented by the diameter of the semicircle in Fig. 3a. Diffusion resistance is modelled using a Warburg resistor (*R*_w_) which is represented in Fig. 3a as a line at 45° increment in the high-frequency range. Finally, the impedance *Z* of a CPE can be described as:

1 $$ Z=\frac{\left(\mathrm{Y}{0}^{-1}\right)}{(jw)^{\upalpha}} $$

Here α is used to describe to which extent the CPE deviates from an ideal capacitor. When *α* = 1 the CPE equals an ideal capacitor. For 0.5 < *α* < 1, capacitive dispersion increases with decreasing *α* values. When α~0.5, the CPE behaves as a Warburg resistor and for 0 < α < 0.5, the CPE approaches resistor behaviour [29, 30]. Figure 3d shows the values for *R*_ct,_ *R*_z′0_, *α* and *R*_w_ from the model fitting data (all *χ*^2^ values < 0.001). *R*_w_ was around 390–400 Ω for all SPEs which indicated the diffusion limit (as expected) remained equal for all SPEs. However, a decrease in frequency dependent impedance in the mid frequency range, indicated a decrease in capacitive behaviour for the nanomaterial-SPEs and, to a lesser extent, for the pre-SPEs (Fig. 3b). This was equally observed by a gradual decrease in *α* from c-SPE to pre-SPE to GNP-SPE to CB-SPE (Fig. 3d). Indeed, *α* values for CB-SPE and GNP-SPE were ~ 0.5 indicating the CPE behaved as a Warburg resistor in those cases. A more than 20-fold decrease in *R*_ct_ for the nanomaterial-SPEs (around 50 Ω) was observed compared with c-SPE (around 1300 Ω) (Fig. 3d). However, *R*_ct_ and *α* values did not differ significantly between CB-SPE and GNP-SPE. Difference between these nanomaterial-SPEs was observed for the *R*_z′0_ values, which doubled for CB-SPE compared with all other SPEs (Fig. 3d). This increase in *R*_z′0_ was due to an increase in intrinsic and/or contact resistance since the electrolyte solution was identical for all EIS measurements. A clear difference between CB-SPE and GNP-SPE was observed in the admittance plot (Fig. 3c). Here the diameter of the semicircle, which represented a parallel combination of conductance and capacitance [31], was smaller for CB-SPE. Thus, according to this EIS analyses, capacitance and *R*_ct_ of both nanomaterial-SPEs were lower than that of pre-SPE and c-SPE. Capacitance and overall interfacial admittance of CB-SPE was lower than for GNP-SPE while *R*_z′0_ was about double its value.

### SEM analyses of modified SPEs

**Fig. 4:**
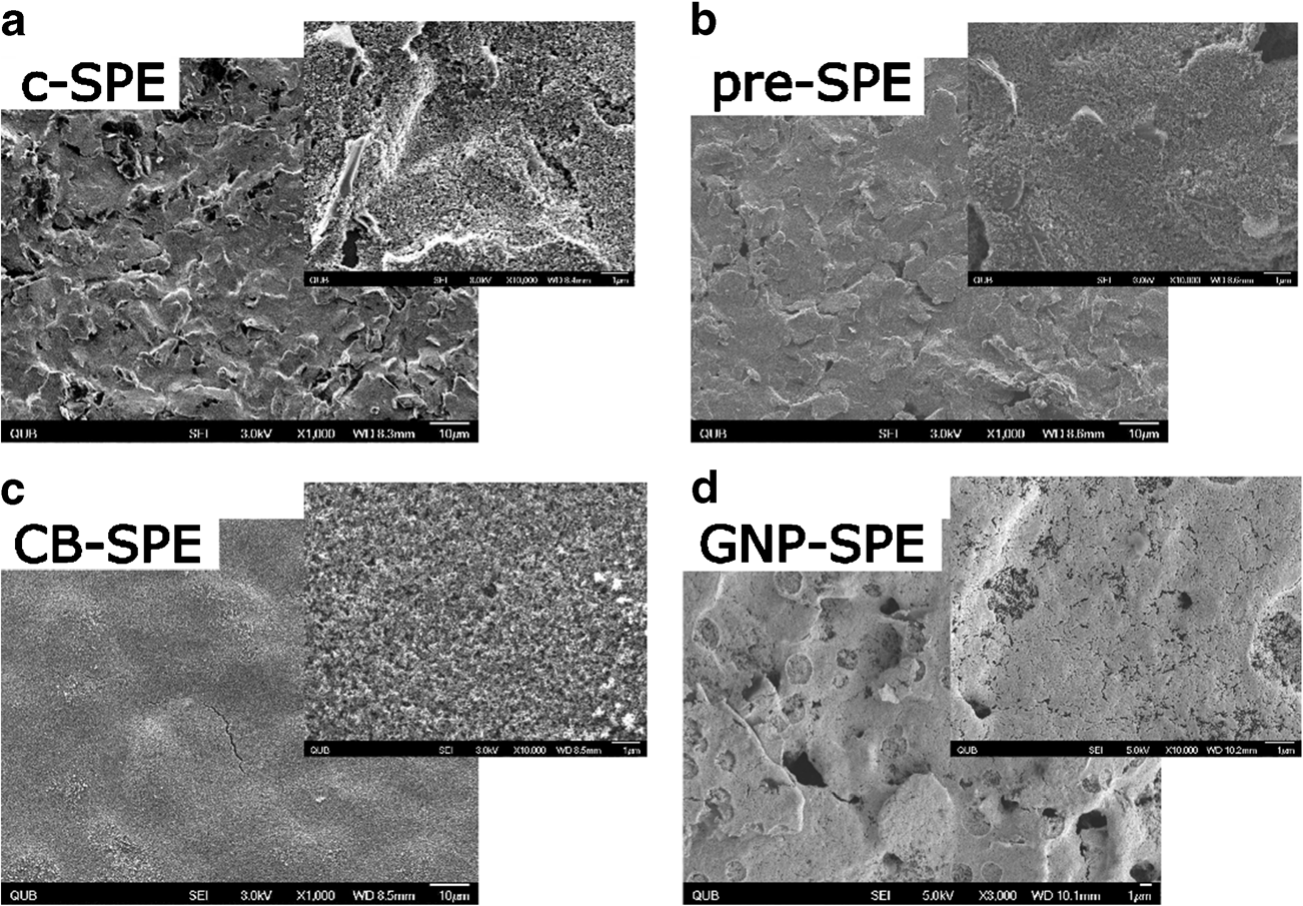
**a** c-SPE. **b** Pre-SPE. **c** CB-SPE. **d** GNP-SPE. All images were taken using a scanning electron microscope set at × 1000 magnification or × 10,000 magnification (inset figures). Scale bars are 10 μM and 1 μM for inset figures and indicated in the right down corner of each image

SEM images (Fig. 4) show that CB-SPE had a smoother, more uniform surface compared with the other SPEs. GNP-SPE and pre-SPE surfaces were also quite homogeneous with pre-SPE featuring less polymeric linker compared with c-SPE while GNP-SPE is covered by a thick, homogenous layer of GNPs. Pockets and cracks were observed on the surface which may account for an increase in capacitance compared with CB-SPE. The changes in porosity observed may have resulted in changes in contact resistance as has been previously observed for other materials [32]. Since CB is a semi-conductor, these changes may explain the higher *R*_z′0_ values for SPE-CB compared with GNP-SPE, which was covered by a conductive material.

### Optimizing DA determination with nanoparticle-SPEs

#### Biosensor development

**Fig. 5:**
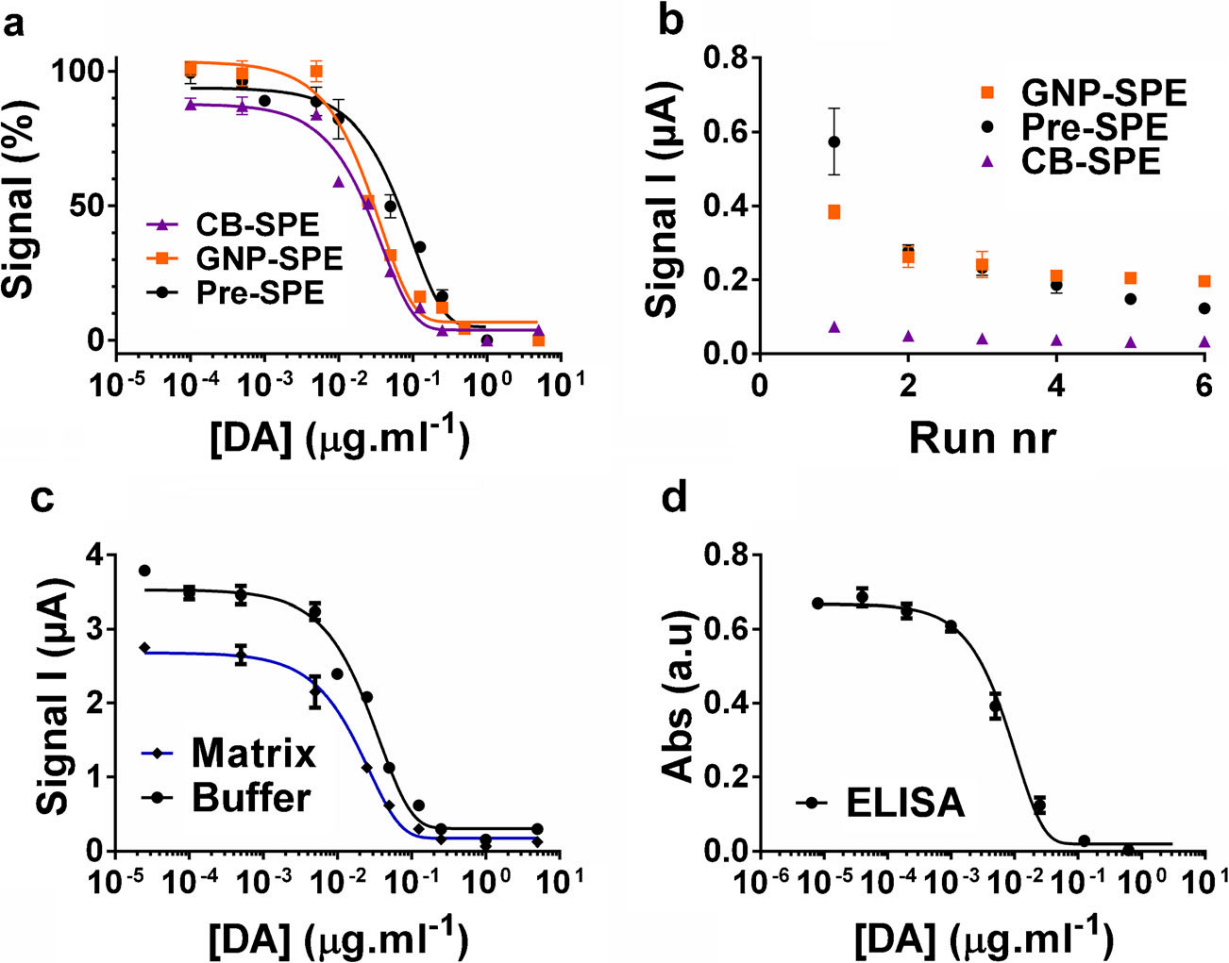
**a** Calibration plots for DA quantification in buffer using pre-SPE (black circles), CB-SPE (purple triangles) or GNP-SPE (orange squares). **b** Signal observed in chronoamperometry experiments on the CB-, GNP- and pre-SPEs in citrate buffer with KCl for 6 consecutive runs. **c** Calibration plots in buffer (black) and matrix (blue) using CB-SPE. **d** Calibration plot for DA determination using the same DA-mAb and BSA-DA reagents in a classic indirect competitive ELISA assay. Reagent concentrations used in the ELIME assay are as follows: DA-mAb 0.075 μg on 75 μg MBs per mL reagent and 3.8 μg mL^−1^ HRP-pAb. In the ELISA assay, DA-mAb conc. was 0.008 μg mL^−1^, BSA-DA 1.25 μg mL^−1^ and HRP-pAb 0.16 μg mL^−1^. *n* = 3 for all experiments. Mean and SD are indicated although SD is not always visible (smaller then points)

The amounts of hapten-coated MBs (3.75–15 μg per chronoamperometry measurement), DA-mAb (0.0375–1 μg mL^−1^) and HRP-pAb (1–7.8 μg mL^−1^) were varied to optimize the ELIME assay. Detailed descriptions and Fig. S9 describing these optimizations can be found in the supplementary material section 3.1.5. Briefly, the following experimental conditions were found to give the best results and were used here: 3.8 μg mL^−1^ HRP-pAb, 0.075 μg mL^−1^ DA-mAb and 7.5 μg MB-DA-BSA per chronoamperometric measurement. GNP-SPE, CB-SPE and pre-SPE were used as transducers for the ELIME assay and create a calibration plot in buffer (Fig. 5a). The fit of the dose-response curves showed excellent *R*^2^ values (0.98 and above) and very little variance between replicates. The lowest LOD (0.4 ng mL^−1^) was observed when CB-SPE was used. Pre-SPE followed with a LOD tenfold higher (4 ng mL^−1^), shortly followed by GNP-SPE with a LOD of 6 ng mL^−1^. For IC_50_ values however GNP-SPE is very close to CB-SPE with 30 versus 23 ng mL^−1^ respectively while pre-SPE has an IC_50_ of 66 ng mL^−1^ (Table 1).

**Table 1:** The analytical parameters of the calibration plots. The 90%, 50% and 20–80% signal values were interpolated from a four-parameter dose-response curve fitted to normalized data to acquire the LOD, IC50 and linear range respectively. Assay type, *R*^2^ value of the curve fit and used SPE modification as well as matrix used are indicated

Assay	Matrix	LOD (ng mL^−1^)	IC_50_ (ng mL^−1^)	Lin. range (ng mL^−1^)	*R*^2^
ELIME pre-SPE	Buffer	4	66	6–172	0.98
ELIME GNP-SPE	Buffer	6	30	11–73	0.98
ELIME CB-SPE	Buffer	0.4	23	5–62	0.98
ELIME CB-SPE	Scallop extract	0.7	21	5–58	0.98
ELISA	Buffer	0.9	8	2–18	0.99

#### Study on background signal

Another difference between GNP-, pre- and CB-SPEs observed was the background current recorded in citrate buffer for multiple sequential runs of 30 s (Fig. 5b). Pre-SPE and GNP-SPE showed a gradual decrease in background current from approx. 0.5–0.4 μA at run 1 to 0.2–0.15 μA at run 6. CB-SPE showed virtually no decrease in background current and stayed well under 0.1 μA from run 1. This is most likely the cause of the better performance of CB-SPE. EIS analyses (Fig. 3) showed that *R*_z′0_ was higher for CB-SPE while overall conductivity and capacitance was lower than for all other SPEs. These features of CB-SPE may explain the observed low background currents. Charging a capacitor in series with a resistance using DC can be described by:

2 $$ I=\frac{V_0}{R\times {e}^{\left(-t/ RC\right)}} $$

where *V*_0_ is applied potential, *R* total resistance, *C* capacitance, *t* elapsed time since potential application and RC is the time constant (which represents the time it takes for a transient current to reach steady state). Thus, if *t* = 0 then *I* = *V*_0_ R^−1^. Thus, the observed lower initial current for CB-SPE can be explained by the higher *R*_z′0_. In addition, low capacitance in CB-SPE likely leads to a reduced time constant compared with all other SPEs leading to a shorter period to reach steady state. These observations concur with observations made from SEM images (Fig. 4) showing that CB is more homogeneously distributed on the WE leading to less pores. This may explain the observed changes in capacitance and contact resistance compared with more porous GNP- and pre-SPE [32] [33]. Thus, CB-SPE was selected as the optimum choice to determine the selectivity of the assay and quantify DA in natural contaminated scallop samples.

#### Study of matrix effect and recovery studies

Samples were extracted and the matrix was diluted × 1000 to obtain IC_50_ levels of the previous buffer curve at the action level for DA (20 mg kg^−1^ of tissue) set in the European Union [34]. When the curves in matrix and buffer were overlaid, it was clear that some matrix effects lead to the reduction in maximum observed current (Fig. 5c). However, only a slight increase in LOD (from 0.4 ng mL^−1^ in buffer to 0.7 ng mL^−1^ extract) occurred while the linear range remained virtually the same (Table 1). Thus, with this assay, an LOD, IC_50_ and linear range of 0.7, 21 and 5–58 mg DA kg^−1^ shellfish were achieved which makes the LOD more than × 25 more sensitive as the required stated action level [34] in shellfish. Furthermore, the analytical parameters of the calibration plot made for shellfish matrix using CB-SPE were superior compared with the parameters of a classic ELISA calibration plot in buffer (Fig. 5d). The analytical parameters of the biosensor were compared with other sensors with varying portability potential (based on the size of the used instruments) in Table 2. This comparison clearly shows that the biosensor reported here outperforms less portable optical methods such as flow cytometry [35] and surface plasmon resonance [22]. However, it does not reach the same sensitivity as LC-MS analysis with selective extraction using a metal–organic framework [37]. This is not surprising due to the high sensitivity of this type of non-portable, laborious LC-MS analyses which is considered the golden standard for DA determination [2] [3]. A fluorescence-based microarray shows similar performance to the CB-SPE biosensor although it was tested in a less demanding matrix (artificial seawater). Additionally, the microarray is less portable due to the reader size [24]. Finally, an electrochemical method using SPEs outperformed the method reported here in sensitivity by a factor 4 [17]. However, this method requires individual SPE bio-functionalization and was only tested in buffer. Next DA amounts in 2 naturally contaminated scallop samples were determined by interpolating signals for these onto the matrix calibration plot. The samples were highly positive containing 47 and 25 μg DA per g shellfish, which is 2.35 and 1.25 times higher than the EU action level. DA concentration in these samples was equally quantified using HPLC [22] and determined to be 53 and 30 μg DA per g shellfish respectively. Thus, the data using this biosensor showed 86% ± 5% agreement (*R*^2^ = 0.965) with HPLC data on average. Overall, the method shows very good sensitivity and does not require lengthy SPE modification procedures with costly nanomaterial or individual SPE bio-functionalization. Additionally, the method has excellent portability potential due to the ever-reducing size of potentiostats (which can even be fitted to smartphones https://www.palmsens.com/product/sensit-smart/). The method was also shown to work well in shellfish matrix. These features make the method highly competitive with other sensors listed in Table 2 and insure a wide scope for its application. An additional advantage is that the working potential of the sensor (− 50 mV) is lowered by a factor of four compared with the potential needed for bare c-SPE sensors (approx. − 200 mV) due to CB modification (see Fig. S3 in supp. section 3.1.2). Because of this excellent characteristic of CB and the outstanding selectivity of the used antibody, the system functions in the presence of a wide range of interferences without being affected. This being said, a limitation of the system is equally identified. Multiplexing this system through use of microfluidics and a multi-channel potentiostat is complicated due to the use of the MB conjugates and magnets under the WE. For such a setup, direct immobilization of the hapten may be more appropriate.

**Table 2:** Analytical parameters of DA determination methods reported in the literature and here. CR is cross-reactivity. Ab is antibody. High-performance liquid chromatography is HPLC. MS is mass spectrometry. BLI is biolayer interferometry. STX is saxitoxin. ANA-a is anatoxin. CYN is cylindrospermopsin. MC-LR is microcystin-LR. STX is saxitoxin. TTX is tetrodotoxin. OA is okadaic acid. GA is glutamic acid. GluNH2 is l-glutamine. AA is l-ascorbic acid. Asp.A is aspartic acid. PDBE is polybrominated diphenyl ether flame retardant

Matrix	Materials used	Anal. method	Analytes	LOD (for DA only)	Specificity (for DA only)	Portability potential	Method compared in study	Ref.
Freshwater and seawater	Ab-conjugated microspheres	Flow cytometry	Saxitoxin, ANA-a, CYN and MC-LR, DA	1.9 μg L^−1^ (not specified)	CR not reported	Low	LC-MS	[35]
Seawater	Fluorophore-conjugated Ab	Fluorescent microarray	DA and various non-related compounds	0.71 μg L^−1^ (artificial seawater)	CR against triazine, sulphonamide, PBDE, chloramphenicol, 17β oestradiol found low	Medium	No	[24]
Shellfish	Ab	Surface plasmon resonance	DA, OA, saxitoxin and palytoxin	4000 μg kg^−1^ shellfish	CR tested and found 0% for other toxins screened	Low	AOAC HPLC method	[22]
Shellfish	Polymer-coated optical fibre/Ab	BLI-immunosensor	Domoic acid	LOD not reported IC50 2 μg L^−1^ (extract)	CR not reported	High	No	[36]
Shellfish	SPEs/Ab	SPEs and differential pulse voltammetry	Domoic acid	5 μg L^−1^ (buffer)	CR not reported	High	ELISA	[18]
Buffer only	SPEs/Ab	SPEs/amperometry	Domoic acid	0.1 μg L^−1^ (buffer)	No CR for AA, GA, geranic acid, 2-methyl-3-butenoic acid	High	ELISA	[17]
Shellfish	Modified metal–organic framework	MOF extraction and HPLC-MS	Domoic acid	0.2 ng L^−1^ (not specified)	Selected reaction monitoring mode	Low	NO	[37]
Shellfish	na	AOAC HPLC reference method	Domoic acid	At least 2.7 mg Kg^−1^ shellfish	Retention time	Low	Inter-laboratory study (*n* = 13)	[38]
Shellfish	CB/SPEs/MBs/Ab	CB-modified SPEs/MB hapten/amperometry	Domoic acid	0.4 μg L^−1^ buffer 0.7 μg L^−1^ extract 700 μg kg^−1^ shellfish	No CR detected for STX, Neo, GTX-2, OA, TTX, GA, GluNH2, Asp.A and AA	High	AOAC HPLC method, ELISA	This method

#### Selectivity study

**Fig. 6:**
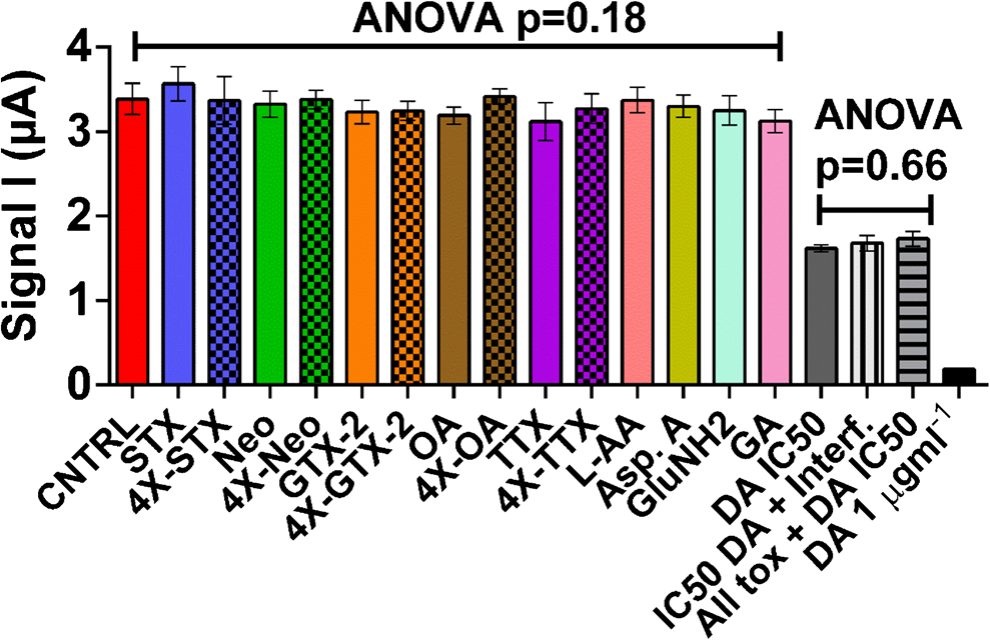
CNTRL shows the chronoamperometry signal with no added DA. STX/4X-STX, Neo/4X-STX, GTX-2/4X-GTX-2, OA/4X-OA and TTX/4X-TTX show the signal (with no added DA) with the toxins spiked at their EU action levels [34] or advised levels (for TTX there is no EU regulated level [39]) and 4X those levels. The concentrations added were 0.8 and 3.2 ng mL^−1^ for STX, NEO and GTX-2; 0.16 and 0.64 ng mL^−1^ for OA and 0.05 and 0.2 ng mL^−1^ for TTX. L-AA, Asp.A, GluNH2 and GA show the signal (with no added DA) with these compounds spiked at 10 μg mL^−1^. DA IC50 shows the signal with 20 ng mL^−1^ DA. IC50 DA + Interf. shows the signal with 20 ng mL^−1^ DA and L-AA, Asp.A, GluNH2 and GA (all at 10 μg mL^−1^). All tox + DA IC50 shows the signal with 20 ng mL^−1^ added DA and all toxins spiked at the highest levels mentioned above. DA 1 μg mL^−1^ shows the signal when 1 μg mL^−1^ DA is added to the blank. *p* values of the one-way ANOVAs comparing all samples with no added DA or 20 ng mL^−1^ added DA are indicated

Selectivity of the CB-SPE biosensor for DA was tested against naturally co-occurring marine toxins (STX, NEO, GTX-2, OA and TTX) as well as the structurally similar compounds GA, GluNH2, Asp.A and L-AA (Fig. 6). One-way ANOVA analyses comparing the signal for the blank (no interferences or DA added) with the signals when the individual compounds were added to the blank at various concentrations (see supplementary material section 2.8) was not significant (*p* = 0.18). Equally, one-way ANOVA comparing the signal when 20 ng mL^−1^ DA was added to the blank with the signals when all the toxins (at 4 times their respective action levels) or all the DA structurally similar compounds (at 10 μg mL^−1^) were added into the solution containing 20 ng mL^−1^ DA was not significant (*p* = 0.66). Thus, the biosensor has good selectivity for DA when compared with these compounds.

## Conclusion

CB-SPE showed a tenfold increase in sensitivity for DA determination compared with pretreated SPEs while analysis times were reduced due to the extremely low background currents observed. However, GNP-SPE also performed well and remains equally interesting if further surface modifications (such as SAM formation of thiol linkers for facile aptamer attachment) are required. Furthermore, the optimum washing conditions using hapten-conjugated MBs enabled the successful development of a biosensor able to quantify DA in naturally contaminated scallop samples with good agreement (*R*^2^ = 0.965) with HPLC data. Overall, this work shows, for the first time, that CB-modified SPEs in combination with an ELIME assay hold great potential for antibody-based determination of low-molecular-weight compounds in a complex matrix. The ease-of-preparation of CB-SPE and the cost-effectiveness make the material an interesting rival nanomaterial for carbon nanotubes and graphene in the race for efficient and sensitive portable nano-biosensors development.

## Electronic supplementary material

ESM 1 (DOCX 1799 kb)
