# Supplementary material for: The benefits of carbon black, gold and magnetic nanomaterials for point-of-harvest electrochemical quantification of domoic acid
Source: Mikrochim Acta. 2020 Feb 12;187(3):164. doi: 10.1007/s00604-020-4150-x (PMC7015955; doi:10.1007/s00604-020-4150-x)
Supplement: Supplementary file 1 — (DOCX 1799 kb) [file 604_2020_4150_MOESM1_ESM.docx]

Electronic Supporting Material
on the Microchimica Acta publication entitled:

**The benefits of carbon black, gold and magnetic nanomaterials for point-of-harvest electrochemical quantification of domoic acid**

***Joost L.D. Nelis^1^, Davide Migliorelli^2^, Safiye Jafari^2^, Silvia Generelli^2^, Javier Lou-Franco^1^, J.-Pablo Salvador^3,4^, M.-Pilar Marco^3,4^, Cuong Cao^1^, Christopher T. Elliott^1^ and Katrina Campbell^1^***

**^1.^ Institute for Global Food Security, School of Biological Sciences, Queen’s University of Belfast, 19 Chlorine Gardens, Belfast, UK BT9 5DL**

**^2.^ CSEM SA, Center Landquart, Bahnhofstrasse 1 Switzerland**

**^3.^ Nanobiotechnology for diagnostics (Nb4D), Institute for Advanced Chemistry of Catalonia (IQAC) of the Spanish Council for Scientific Research (CSIC), Jordi Girona 18-26, 08034 Barcelona, Spain**

**^4.^ CIBER de Bioingeniería, Biomateriales y Nanomedicina (CIBER-BBN)**

## 2. Supplementary material and methods:

## 2.1 Apparatus, electrodes, chemicals and software

Ammonium sulfate, TWEEN® 20, Phosphate buffer saline tablets (PBS), HAuCl_4_, L-Ascorbic acid (L-AA), anti-IgG horse radish peroxidase conjugated polyclonal antibody (HRP-pAb), Sodium Citrate, HNO_3 ­_(70%), HCl (37%), NaOH, H_2_SO_4_, H_2_O_2_ (30%), K_4_Fe(CN)_6_·3H_2_O, K_3_Fe(CN)_6_, CaSO_4_, citric acid, KCl, Na_2_HPO_4,_ N,N'-Dicyclohexylcarbodiimide (DCC), N-hydroxysuccinimide (NHS), anhydrous sodium borate, sodium carbonate decahydrate, 3,3′,5,5′-tetramethylbenzidine (TMB), domoic acid (DA), boric acid, bovine serum albumin (BSA) L-glutamic acid, L-glutamine and aspartic acid were purchased from Sigma Aldrich (Irvine; UK; <https://www.sigmaaldrich.com/united-kingdom.html>) and used without further purification. Dimethylformamide (DMF) (Sigma Aldrich) was freshly prepared using CaSO_4_ desiccant powder and vacuum distillation. Monoclonal anti-DA mouse antibodies (DA-mAb) were produced in-house and previously characterized [1] [2]. Monoclonal antibodies were chosen as primary antibody ensuring high specificity while polyclonal antibodies coupled to horseradish peroxidase (HRP-pAb) were used as secondary antibody to ensure multiple binding sites and signal enhancement. CB (N220) was obtained from Cabot Corporation (Ravenna; Italy; <http://www.cabotcorp.com/>). Magnetic beads were obtained from ThermoFisher Scientific (MyOne Dynabeads; <https://www.thermofisher.com/uk/en/home.html>). All EC measurements were performed with a PalmSens 4 potentiostat (Palmsens; The Netherlands; <https://www.palmsens.com/>). Saxitoxin, neosaxitoxin, okadaic acid, and gonyautoxin-2 were purchased from the National Research Council Canada (<https://nrc.canada.ca/en>). Optical absorption measurements were performed using a Cary 60 UV-Vis spectrometer (Agilent; Stockport; UK; <https://www.agilent.com/>). Screen printed electrodes were produced in-house using a semi-automatic screen-printing machine (DEK 248; Wilton; UK; <https://www.uk-cpi.com/>), flexible polyester sheets (Autostat CT; Wantage, UK; <http://autotype.macdermid.com/>) and graphite-, silver- and dielectric-ink (Gwent, Pontypool, UK; <http://www.gwent.org/>). The WE had a geometric area of 0.07 cm^2^. Transmission electron microscopy (TEM) was performed using a JEOL JEM-1400 and scanning electron microscopy (SEM) with a JEOL 6500 (JEOL; UK; Welwyn Garden City; https://www.jeolusa.com). For Palmsens data PSTrace v5.5. software was used. For EIS data fitting Z-view software (Scribner Associates, Inc., Southern Pines, NC, USA; [www.scribner.com](http://www.scribner.com)) was used. Nanoparticle sizes were determined using ImageJ v1.41 (<https://imagej.nih.gov/ij/>). For UV-VIS data Cary WinUV v5.0 was used. Calibration curve normalization and fitting as well as statistical analyses were performed in Graphpad v6.0 (San Diego; CA; USA; <https://www.graphpad.com/>). Normalised calibration curves were fitted using the four-parameter dose-response curve. LOD, IC50 and linear range were obtained by interpolating 90%, 50% and 20-80% signal values from the fitted normalised curves respectively.

## Magnetic bead coating

Five mg of tosyl-activated MBs were resuspended in 200 μL of coating buffer (sodium borate buffer (0.1 M; pH 9.5) and placed in a magnetic rack for 1 min. The supernatant was discarded and 227 μL of coating buffer added. 200 μg of BSA-DA in PBS (pH 7.4) was added followed by an addition of 223 μL of sodium borate buffer (0.1 M; pH 9.5) containing 3 M ammonium sulphate. The mixture was incubated for 24 hr at 37 ^°^C with slow mixing and placed 2 min in a magnetic rack and then the supernatant was discarded. 650 μL of blocking buffer (PBS with 0.5% BSA and 0.05% Tween 20; pH 7.4) was added and incubated (24 hr; 37 ^0^C) at slow mixing. The vial was placed in a magnetic rack for 2 min and the supernatant discarded. Next MBs were washed 3 times with PBS (pH 7.4) containing 0.1% BSA and 0.05% Tween (PBS-Wash). Finally, MBs were resuspended in 200 μL of PBS-Wash (PBS pH 7.4 with 0.1% BSA and 0.05% Tween 20) with final concentration of 25 mg MBs.mL^-1^ with approximately 40 μg of BSA-DA.mg^-1^ of MB. The same procedure was followed to coat the MBs with BSA used as a negative control.

## Bioconjugate preparation (DA-BSA)

DA (1 mg) was dissolved in 100 μL of freshly prepared, dry DMF. 2.5 and 5 molar eq. of NHS and DCC were individually dissolved in 50 μL dry DMF and mixed with the DMF DA solution using slow stirring for 4 hr at room temperature (RT). Next the mixture was centrifuged (1000 rcf, 10 min, 20 °C) and the supernatant was added drop wise to a stirred solution of 0.9 mL of borax buffer (pH 8.7) containing 5 mg of BSA and left to react 4 hr at RT. Next the bioconjugate was dialyzed, lyophilized and stored at -80 °C [3]. Toxin density on the bioconjugate was analyzed using matrix assisted laser desorption/ionization mass spectrometry (MALDI-MS). Briefly, 1 µL of matrix (sinapinic acid (SA) 10mg.mL^-1^ in acetonitrile (ACN), H_2_O and formic acid (FA) (70/30/0.1 % respectively) was pipetted on a steel sample support and left to dry on the bench (20 min). Next 1µL of the sample in ACN/H_2_O/FA (50/50/0.1%) solution was deposited and left to dry on the bench for 20 min. Finally a second layer of matrix (1 µL) was pipetted onto the sample. The toxin density on the BSA conjugate was calculated according to the following equation: found to be to ~ 16 DA molecules per BSA molecule (fig. S1) using the following equation:

$\left[ MW\left( conjugate \right)-MW\left( protein \right) \right]/MW(hapten)$ (1)

With MW being the average molecular weight of the molecule.

***
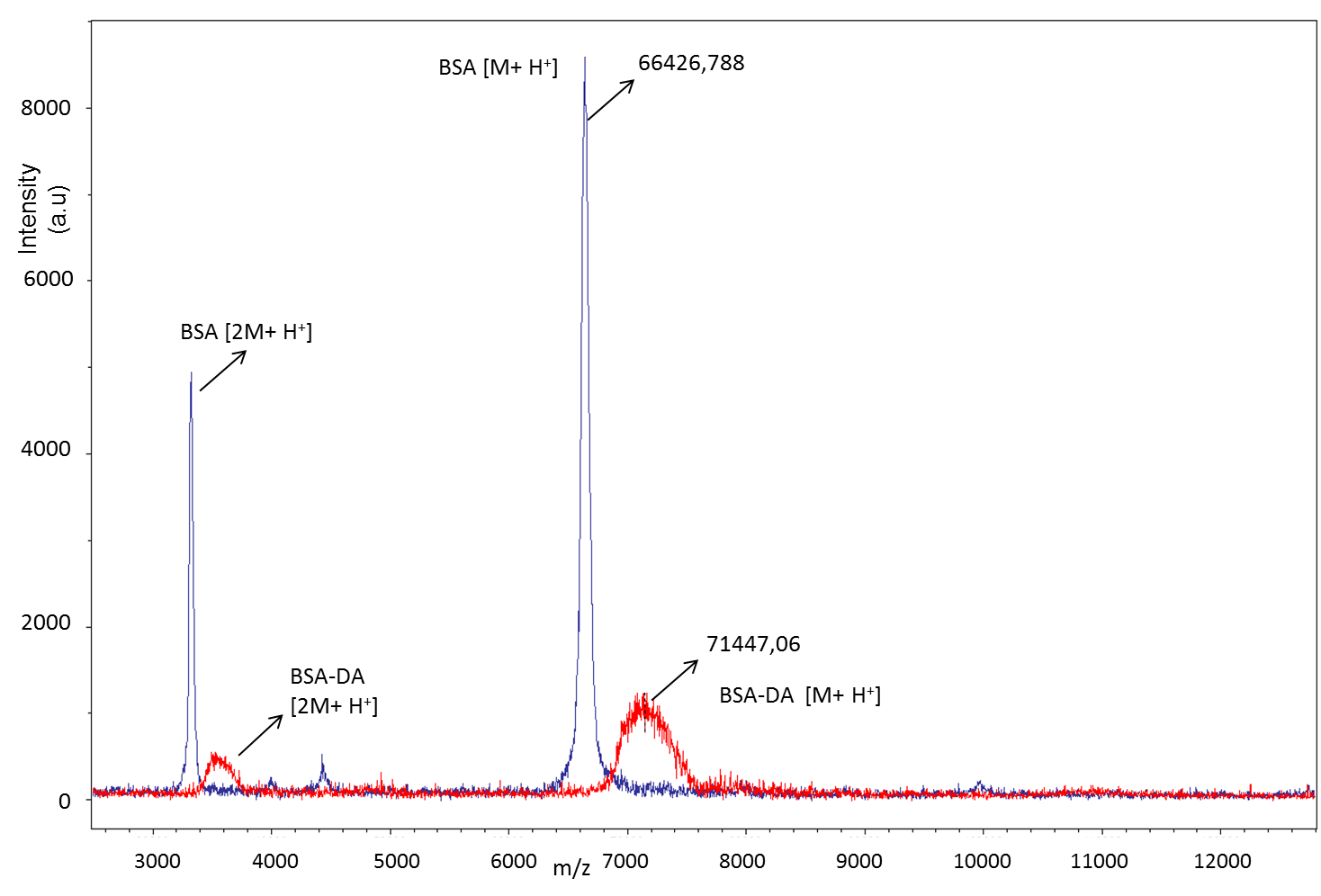
***

***Fig. S1: MALDI-TOF spectra of BSA and BSA-DA.*** *The accumulated spectra of bovine albumin serum protein (BSA; (blue) and BSA conjugated with domoic acid (BSA-DA; red) are shown. Average mass (in Da) of the pseudomolecular ions of both compounds is indicated. Domoic acid density per BSA molecule is estimated to be the ~ 16 using the following formula: (MW BSA-DA) – (MW BSA) / (MW DA) = DA density*

## c-SPE production, modification and CB dispersions

## c-SPE production and ink modification

For c-SPE production the reference electrode was printed with silver/silver chloride ink while the WE and counter electrode were printed with graphite ink. Polymeric Dielectric ink was used to insulate the electrodes and confine the WE. In between each printing step electrodes were cured in an oven. SPEs with GNST or GNP mixed in the ink (i-GNST-SPE and i-GNP-SPE) were prepared by mixing colloidal gold particles (2 nM and 80 nM for both i-GNST-SPE and i-GNP-SPE ink mixtures) with ethylene glycol (1:1). This mixture was added to graphite ink (1:11) respectively, and homogenized manually for 10 min. A negative control (dil-SPE) was performed by mixing a (1:1) water: ethylene glycol with graphite ink (1:11) respectively. All ratios mentioned in this work were volumetric.

## CB dispersions used for SPE modification by dropcasting

For SPE modification with CB 1 and 2 mg.mL^-1^ CB dispersions were made in DMF:H_2_O (1:1) by sonification with a Hielscher’s UP 200ST. For the 1 mg.mL^-1^ dispersion 3 µL; 5 µL; 2 x 5 µL and 3 x 5 µL was casted on the WE. For the 2 mg.mL^-1^ dispersion the casts on the WE were: 1.5 µL; 2.5 µL; 5 µL; 5 µL + 2.5 µL = 7.5 µL and 2 x 5 µL. Thus total amount of CB casted on WEs was: 3, 5, 10 and 15 µg using the 1mg.mL^-1^ CB dispersion and 3, 5, 10, 15 and 20 µg using the 2mg.mL^-1^ CB dispersion.

## 2.7.1 Nanoparticle synthesis

Prior to synthesis, glassware was consecutively cleaned with piranha and aqua regia solution to remove organic and inorganic residues. GNPs synthesis followed the Turkevich method [4]. Briefly, 500 µL of 100 mM HAuCl_4_ was added to 194.5 mL MQ and brought to boil at high stir velocity in a round bottom flask equipped with a condenser. Next 5 mL sodium citrate solution (1% (w/v)), was added in 1 shot and the mixture was left to boil for 30 minutes then cooled down gradually and formed the stock solution (2.8 nM) which was stored at 4 ^°^C. Finally a 40X concentration was obtained by centrifuging (13000 RCF; 30’; 20 ^°^C). Concentrates were used to create SPEs with various GNP concentrations (in ink and by drop casting). For GNST synthesis 100 μL of unconcentrated GNPs solution was added to a 0.25 mM HAuCl_4_ solution (10 mL) containing 10 μL of HCl (1 N) in a glass vial under moderate stirring. Next, 100 μL of 3 mM AgNO_3_ and 50 μL of L-AA were added causing a quick colour change from light red to blue/green and left to react for 2’ after which sodium citrate (3 mM final conc.) was added. This mixture was left to agitate overnight after which it was centrifuged (1200 RCF; 25’; 10 ^°^C), and the pellet resuspended in 7.5 mL MQ to reach a stock solution at 2.8 nM, which was stored at 4 ^°^C. Further concentration (40X) was performed immediately prior to creating the modified SPEs to limit aggregation issues. Finally, the GNP concentration was estimated using a protocol detailed in [5] while GNST concentration was estimated following a method detailed in [6].

## Electron microscopy

For top morphology imaging of the SPEs a Field-emission gun Scanning electron microscope (FEG-SEM) equipped with an energy dispersive spectrometer (JEOL 6500) was used which has an ultimate resolution of 1.5 nm and operates at 0.5 to 30 kV and has a magnification range of 10x to 400,000x. Samples were mounted on aluminium discs by using cooper tape for conduction purposes. Imaging was achieved at lower energy (3 kV) and small currents in order to prevent charging effects. The Transmission Electron Microscope (TEM) was a Jeol JEM-1400 Plus equipped with JEOL ‘Ruby’ 8MP Bottom Mounted CCD Digital Camera (JEOL, U.K.). This TEM can chose from 30-120 kV accelerating voltage and magnification between 10x and 1million x and has 0.32 nm ultimate resolution. For TEM analysis samples were prepared on a formvar carbon mesh (Agar scientific). 120kV was used and various magnifications applied to visualize the particles.

## ELISA assays

A standard indirect competitive ELISA protocol for small molecules, as previously described [7], was optimized and used. Briefly, microtiter plates were coated with (1.25 µg.mL^-1^) BSA-DA in carbonate buffer (100 μL per well; overnight; 4 °C). Next plates were washed four times with PBST. PBST solutions containing various amounts of DA were then added to the plates (50 μL per well), followed by the addition of PBST containing an optimized amount (0.008 µg.mL^-1^) DA-mAb (50 μL per well) and incubated (30’; RT). The plates were washed as before, and incubated with 100 μL per well HRP-pAb in PBST (0.16 µg.mL^-1^) for 30 min at RT. After an additional washing step, 100 μL per well Citrate buffer containing 0.01% H_2_O_2_ and 3% TMB (pH 5.5) was added. The enzymatic reaction was stopped after 30 min at RT with 4 N H_2_SO_4_ (50 μL per well) and absorbance read at 450 nm.

## 2.8 Selectivity

Selectivity of the biosensor for DA against saxitoxin (STX), neosaxitoxin (NEO), gonyautoxin-2 (GTX-2), okadaic acid (OA) and tetrodotoxin (TTX) was tested. For these experiments the signal obtained with the optimised ELIME assay using CB-SPEs in buffer (containing no DA) was compared (one-way ANOVA) with the signal obtained when the following toxins were individually added: saxitoxin (STX), neosaxitoxin (NEO), gonyautoxin-2 (GTX-2), okadaic acid (OA) and tetrodotoxin (TTX). The concentrations added were: 0.8 and 3.2 ng.ml^-1^ for STX, NEO and GTX-2, 0.16 and 0.64 ng.ml^-1^ for OA and 0.05 and 0.2 ng.ml^-1^ for TTX. These concentrations represent the EU regulated action levels [8] or advised levels (for TTX since there is no regulated level [9]) and a 4X increase of those levels (taking the dilution factor used to get the action level of DA at the IC_50_ of the calibration curve into account). Additionally, the compounds L-glutamic acid (GA), L-glutamine (GluNH2), aspartic acid (Asp.A) and L-ascorbic acid (L-AA) that have structural similarity to DA and are usually present in shellfish tissue [2], [10] were tested for their interference as well. The signal obtained with the optimised ELIME assay using CB-SPEs in buffer (containing no DA) was compared (one-way ANOVA) with the signal obtained when these compounds were spiked at 10 µgml^-1^ following the concentrations used in [2]. To further test for cross-reactivity all the toxins mentioned above were mixed (at the highest mentioned levels above) together with 20 ng.ml^-1^ DA (IC_50_). Equally, 10 µg.ml^-1^ of GA, GluNH2, Asp.A and L-AA was mixed with 20 ng.ml^-1^ DA. The signal obtained with these mixes was compared (one-way ANOVA) with the signal obtained when only 20 ng.ml^-1^ DA was added to the solution.

## 3.1 OPTIMIZATION STUDIES

### 3.1.1 Nanoparticle synthesis

Synthesis of GNPs was confirmed using UV-VIS and TEM analyses (fig. S2). Plasmon peaks for GNP and GNST were found at 518 and 752 nm respectively. ImageJ analyses of TEM images resulted in size estimations of 15.1 ± 1.7 nm for GNP and 105 ± 29 nm for GNST particles (n = 100). Slightly smaller, although not significantly different, values (98 ± 19 nm) were obtained by Dynamic Light Scattering for GNST. Despite measuring the hydrodynamic diameter, only spherical particles can be measured accurately, which explains a larger tip-to-tip average diameter measured by imageJ for GNSTs. For GNPs the hydrodynamic size was as previously reported (13.9 ± 2.9 nm) [11].

*
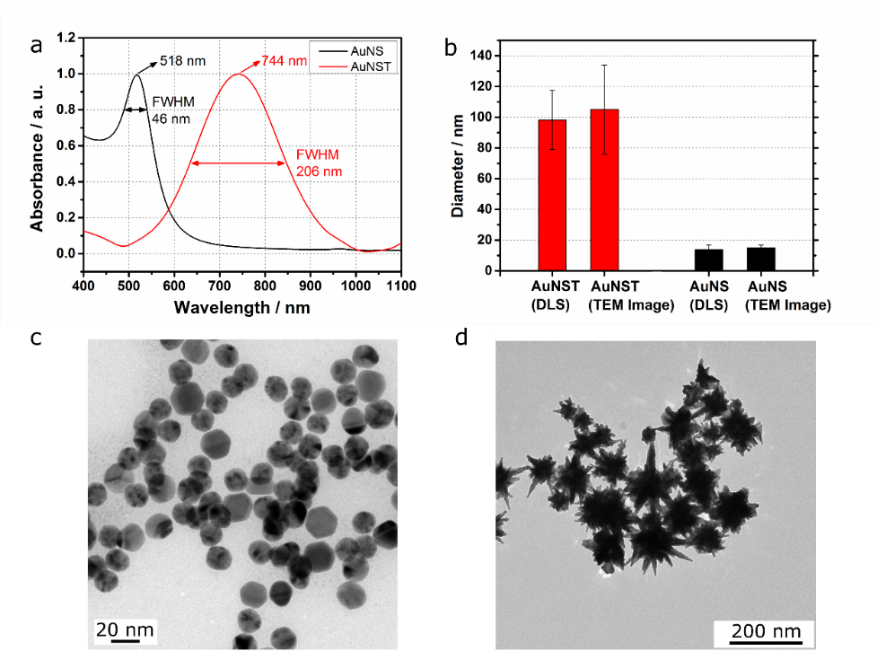
*

***Fig. S2: GNP and NST characterisation.*** *a) UV-Vis spectra of synthesized GNPs and GNSTs. Full width half maximum (FWHM), calculated by fitting to a Gaussian curve, and wavelength at plasmon peaks are indicated. b) The hydrodynamic diameter of the nanoparticles as determined using a Malvern Zetasizer (Nano ZS) and size estimations determined by manual measurements in ImageJ (n=100) from images as shown in c and d. The images were obtained using a JEOL JEM-1400 Plus transmission electron microscope at an operating voltage of 120 kV. Both scale bars represent 200 nm.*

### 3.1.2 Carbon black SPE modification

A 1 and 2 mg.mL^-1^ dispersion of CB was used to acquire SPEs with 3,5,10, 15 and 20 µg CB drop casted onto the WE using 1 to 3 casting steps. CV spectra were obtained (fig. S3) and peak currents as well as ΔE values were extracted (n=3) to determine optimum use of CB for SPE modification. ANOVA analyses showed electro-catalytic activity was significantly different between various CB modified SPEs for both dispersions (p<0.0001) (fig. S4a-b). No significant differences were observed in peak current height between SPEs modified with the same absolute amount of CB using the different dispersions (fig. S4e). An increase in deposited layers however did have a negative effect on reversibility (fig. S4c-d and f). This caused a trade-off between electro-catalytic activity increase, reversibility and number of layers needed which was probably caused by increasing capacitive currents due to multiple layer deposition. Thus use of the 2 mg.mL^-1^ dispersion was preferred to limit the amount of deposition layers needed and gain in reversibility performance and production time. Moreover, no significant differences were observed in electro-catalytic activity for 10 or 15 µg of CB. Thus, use of 10 µg of CB was used from hereon since this limits material use and requires only 1 casting step.

*

*

***Fig. S3: CVs of CB modified SPEs.*** *Left: Cyclic voltammogram (CV) in [Fe(CN) _6_]^3-/4-^ of SPEs modified with 10 µg CB (purple line) or bare c-SPEs (black lines). Right CV of same SPEs in Phosphate buffer with KCl.*

*

*

***Fig. S4: Optimization of CB SPE modification.*** *Statistical analysis of differences in peak current and redox reversibility for redox reactions in [Fe (CN) 6]^3-/4-^ in function of casted amounts of CB using either 1 or 2 µg.mL^-1^ CB dispersions. Absolute amounts of CB casted on the WEs are indicated. 0 µg CB was c-SPE. Peak currents and ΔE values were extracted from CV spectra (n=3) after which parametric ANOVA analyses were performed. Stars indicate p values as follows: * = p<0.05, **=p<0.01, ***=p<0.001, ****= p<0.0001.*

### 3.1.3 Gold nanosphere and gold nanostar SPE modification

Characterisation of GNP and GNST modified SPEs by EIS analysis (fig. S5a) showed a decrease in charge transfer resistance (R_ct_) presented by the diameter of the semi-circle in the Nyquist plot, in function of GNP and GNST concentration. Statistical analysis (t-test) on the performance of SPEs modified with the highest concentrations of GNP and GNSP showed that electrochemical activity was highest for GNP-SPE (p<0.01) while reversibility was equal for both (fig. S5b). SEM analyses for GNST-SPE (fig. S6) showed clear nanoparticle aggregations with spots of visible graphite background. Thus GNST aggregation appeared to have occurred which can account for the lower electro-catalytic activity.

***
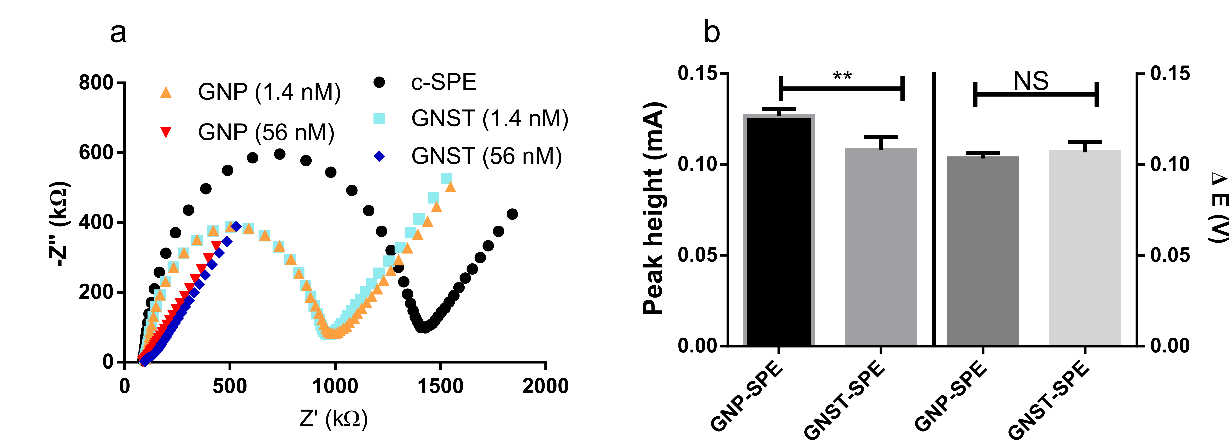
***

***Fig. S5: EIS and CV analyses of GNP and GNST modified SPEs.*** *a) Nyquist complex impedance plot in [Fe(CN)_6_]^3-/4-^ of the GNP-SPEs and GNST-SPEs at the concentrations indicated in the index. b) Bar chart comparing peak height (left y-ax) and redox reversibility (right y-ax) of CVs shown in fig. 2 of the main text (n=3). Significance of results of one tailed t-tests is shown (** equals p<0.01). Voltage sweep was between -0.3 and 0.6 V step rate was 50mV.s^-1^.*

*
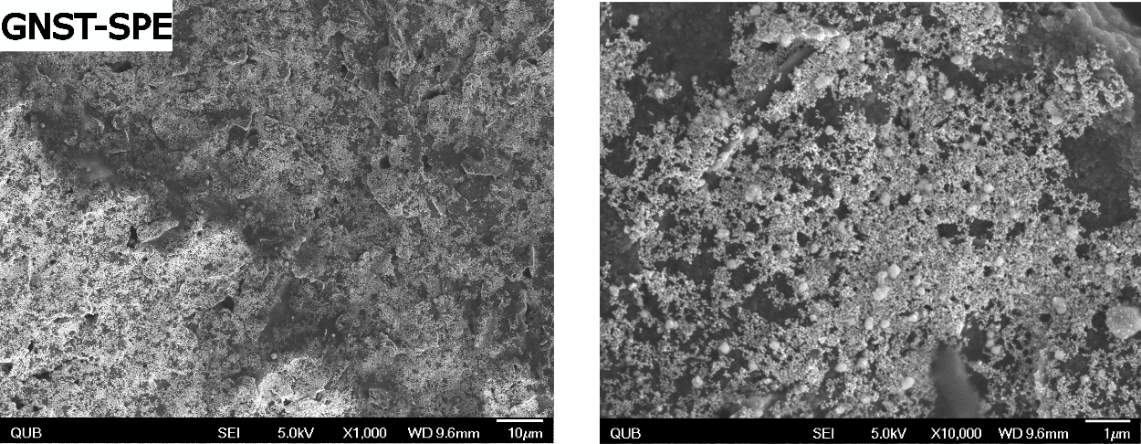
*

***Fig. S6: SEM images of GNST-SPE.*** *Images were taken using a scanning electron microscope set at 1000X magnification (left) or 10,000X magnification (right). Scale bares are 10 µM and 1 µM for inset figures and indicated in the right down corner of each image.*

### 3.1.4 Comparison of nanomaterial-SPE sensitivity for HQ reduction using chronoamperometry

The active surface area (A_active_) of GNP-SPE and CB-SPEs at various concentrations was calculated. To this end the Randle-Sevcik equation for reversible redox processes was used.

$I=2.69\times{10}^{5}\times\sqrt{n^{3}}\times\sqrt{D}\times C_{0}\times A_{active}\times\sqrt{V}$ (1)

I is the current (in Ampere), n, electron transfer per reaction (1 in the case of [Fe(CN)_6_]^3-/4-^), D, diffusion coefficient of [Fe(CN)_6_]^3-/4-^ (7.6 10-6 cm^2^.s^-1^), C_0_, the concentration of [Fe(CN)_6_]^3-/4-^ (0.005M) and V the potential applied (in volt). If I is plotted against$\sqrt{V}$, using different scan rates, a linear curve is obtained with a slope equal to $\frac{I}{\sqrt{V}}$ which allows to calculate A_active_. This was performed for all CB casts on SPEs and for GNP-SPE (fig. S7a). A_active_ increased in function of added amount of CB and doubled at the highest concentration of CB compared to c-SPE. However, between 10 and 15 µg of CB little increase was observed. As for GNP-SPE, A_active_ was similar to the higher CB concentrations used. Calibration curves for HQ oxidation for the various SPEs were then obtained. Linear regression lines had R^2^ > 0.97 in all cases (fig. S7b). Slope analyses of the regression curves for 10 and 15 µg of CB modified SPEs and GNP-SPE showed no statistical difference for slope (p=0.25) and intercept p=0.32 (Prism linear regression analysis). Thus the small increase of A_active_ observed for the 15 µg of CB modification did not contribute to a significant increase in sensitivity for HQ oxidation. However, a clear increase in slope was observed (p<0.0001) when the latter three nanomaterial-SPEs were compared to c-SPE and SPEs using lower amounts of CB. Thus, CB has a catalytic effect on the redox couple HQ/BQ. However, if the cause of this increased catalytic effect was due to the catalytic effect of CB or just to an A_active_ increase of the surface area could not be determined with this characterization. Thus, correlation between A_active_ and catalytic activity for HQ oxidation was investigated. Here a ratio R was calculated by dividing the slopes of the HQ oxidation curves of the various modified SPEs in fig. S7b by the corresponding active surface areas of those SPEs. The ratio was plotted against increasing amounts of CB. The points were fitted against a one phase decay function which showed high correlation (R^2^=0.98) (fig. S7b inset). Such a correlation would not be expected if the increase in HQ oxidation sensitivity was partly due to factors other than A_active_ increase. Moreover, further increase of A_active_, than that calculated for the 10 µg CB modification, will most likely not result in an increase of catalytic activity for HQ oxidation since GNP-SPE, and the 10 and 15 µg of CB-SPEs are close to the asymptote of the function in fig. S7b inset. Thus only GNP-SPE and the SPE modified with 10 µg of CB (called CB-SPE from hereon) were used in further experiments.

*
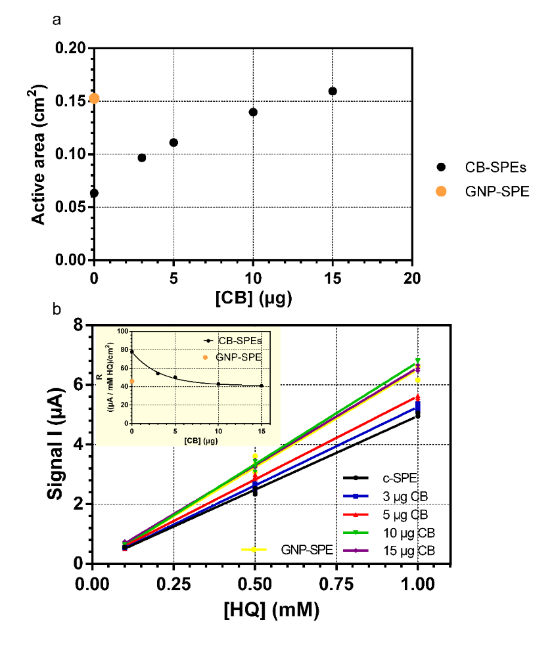
*

***Fig. S7: Active surface area characterisation.*** *a) Active surface area calculated for SPEs with various amounts of CB casted on the WE as well as for GNP-SPE. CV experiments were ran at different scan rates and the surface area calculated by using the Randle-Sevcik equation. b) The sensitivity of c-SPE, CB-SPEs at various concentrations of CB and GNP-SPE towards the oxidation of hydroquinone (HQ) (from 0.1 to 1 mM HQ) determined by chronoamperometry. (n=3). Individual points are shown. Ratio of the slopes from linear regression lines shown in (b) divided by corresponding active surface area shown in (a) plotted against CB concentration are shown in inset figure. Position of GNP-SPE ratio is equally shown. Exponential decay was assumed for line fitting. R^2^ was 0.98.*

***3.1.4 Optimization of SPE pretreatment procedure***

The pretreatment procedure was optimized as optimum pretreatment parameters (time and amount of voltage applied) are ink specific [12]. Either +1.7 or +1.5 volts were applied to the WE for 30 to 180 sec in PB-KCl. These measurements showed irregular current signal when 1.7 V was applied for more than 50 sec, indicating damage to the SPE (fig. S8a). Thus, only 30 s and 50 s pretreatments were further studied. For 1.5 V, limited current increase was observed indicating less redox reactions occurring at the electrode surface when compared to 1.7 V. However, both treatments increased electro-catalytic activity compared to untreated electrodes (fig. S8b). Finally EIS analyses were performed (fig. S8c) and data was fitted to a modified Randles circuit (capacitor was replaced by a constant-phase-element (CPE)), to estimate equivalent circuit parameters (fig. S8d). 30s and 50s pretreatment times resulted in R_ct_ values around 400 Ω using 1.7V and around 700 Ω for 1.5V. However, no significant difference was detected between 30 and 50 seconds if 1.7 V was applied. Thus 30s using 1.7V was selected from hereon.

*
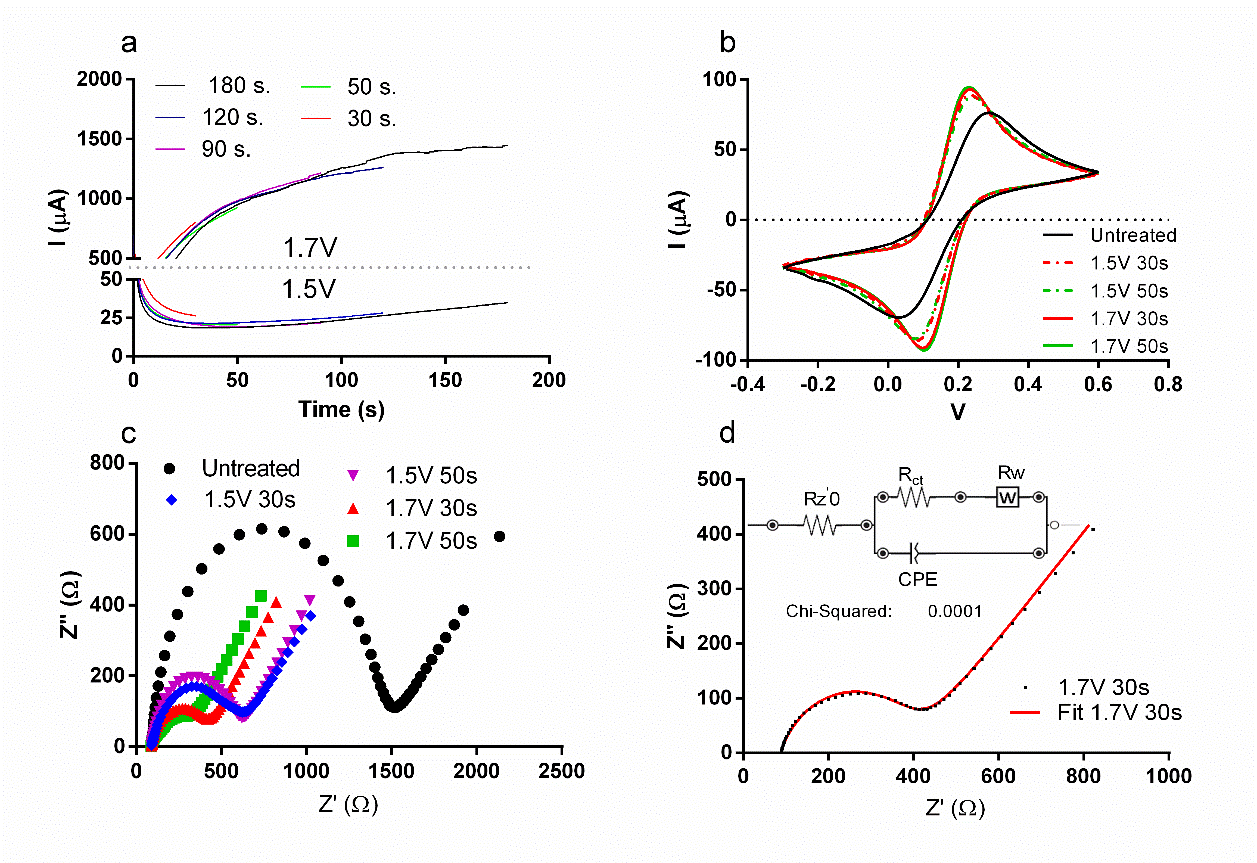
*

***Fig. S8: Pre-treatment optimization.*** *a) Chronamperometric measurements recorded during pretreatment using 1.7V (up the grey dashed line) or 1.5V (below grey dashed line). Colours indicated time voltage was applied as in legend. b) Cyclic voltammograms of SPEs pretreated for 50 (green) or 30 (red) seconds using 1.5 (broken lines) or 1.7 (full lines) volt. Black line represents untreated c-SPE c) electrochemical impedance spectroscopy measurements on the SPEs shown in (c). Pretreatment time and voltage is as indicated in legend. d) Example of fitting the EIS data (black dots) to the equivalent circuit model (red line) used throughout the article (modified Randles circuit). Data set used in (d) was a SPE pretreated for 30 s. at 1.7 V. Chi2 value of the fit is indicated. Rz’0 represents the sum of electrolyte, intrinsic material and contact resistance, Rct charge transfer resistance, Rw Warburg resistor, CPE constant phase element*

### 3.1.5 Optimization of immunoreagents used

The amounts of hapten coated MBs (3.75-15 µg per chronoamperometry measurement), DA-mAb (0.0375-1 µg.mL^-1^) and HRP-pAb (1-7.8 µg.mL^-1^) were varied to optimize the ELIME assay. For each parameter chronoamperometry signals were recorded for MB-BSA-DA and MB-BSA conjugates (the latter serving as negative control) (fig. S9). The highest signal to noise (S/N) ratio for HRP-pAb was obtained at 3.8 µg.mL^-1^ (fig. S9a) and is used from hereon. Next MB amount and DA-mAb concentration were optimized (fig. S9b). Highest S/N ratio was observed using 7.5 µg DA-BSA coated MBs per chronoamperometric measurement and 0.1 µg.mL^-1^DA-mAb. Further optimization of DA-mAb concentration was performed using calibration curves in buffer using 0.05, 0.075 or 0.1 µg.mL^-1^DA-mAb (fig. S9c). This identified 0.075 µg.mL^-1^DA-mAb as the most appropriate concentration in terms of LOD and IC_50_. Concentrations of 0.075 µg.mL^-1^DA-mAb, 7.5 µg MB-DA-BSA per chronoamperometric measurement and 3.8 µg.mL^-1^HRP-pAb were used in further experiments.

***

***

***Fig. S9: Optimization of the ELIME assay.*** *a) Optimisation of HRP-pAb concentration using 0.2 µg.mL^-1^DA-mAb or 30 µg.mL^-1^BSA-DA. b) Optimisation of MB-DA-mAb concentration using 3.8 µg.mL^-1^HRP-pAb and MBs coated with 40* *µg BSA-DA per mg MB (or BSA for negative control). Amount of MBs used per single chronoamperometry measurement was varied between 15, 7.5 and 3.75 µg. c) Calibration curves for DA using 3.8 µg.mL^-1^HRP-pAb, 7.5 µg MBs coated with* *BSA-DA (40 µg.mg^-1^ MB) and 0.05 µg.mL^-1^DA-mAb (circles), 0.075 µg.mL^-1^DA-mAb (triangles) and 0.1 µg.mL^-1^DA-mAb (squares). n=3 for all experiments. Mean and SD are indicated although SD is not always visible (smaller than points). Pre-SPEs were used for all experiments.*

**References:**

1. Campbell K, McNamee SE, Huet A-C, Delahaut P, Vilarino N, Botana LM, Poli M, Elliott CT (2014) Evolving to the optoelectronic mouse for phycotoxin analysis in shellfish. Anal Bioanal Chem 406:6867–6881 . doi: 10.1007/s00216-014-8156-2

2. Yakes BJ, Buijs J, Elliott CT, Campbell K (2016) Surface plasmon resonance biosensing: Approaches for screening and characterising antibodies for food diagnostics. Talanta 156–157:55–63 . doi: 10.1016/j.talanta.2016.05.008

3. Sanchis A, Salvador J-P, Campbell K, Elliott CT, Shelver WL, Li QX, Marco M-P (2018) Fluorescent microarray for multiplexed quantification of environmental contaminants in seawater samples. Talanta 184:499–506 . doi: https://doi.org/10.1016/j.talanta.2018.03.036

4. Turkevich J (1985) Colloidal gold. Part I. Gold Bull 18:125–131 . doi: 10.1007/BF03214694

5. Haiss W, Thanh NTK, Aveyard J, Fernig DG (2007) Determination of Size and Concentration of Gold Nanoparticles from UV. Anal Chem 79:4215–4221 . doi: 10.1021/ac0702084

6. De Puig H, Tam JO, Yen CW, Gehrke L, Hamad-Schifferli K (2015) Extinction Coefficient of Gold Nanostars. J Phys Chem C 119:17408–17415 . doi: 10.1021/acs.jpcc.5b03624

7. Tort N, Salvador J-P, Marco M-P (2012) Multiplexed immunoassay to detect anabolic androgenic steroids in human serum. Anal Bioanal Chem 403:1361–1371 . doi: 10.1007/s00216-012-5904-z

8. The European Parliament and the Council of the European Union (2004) Regulation (EC) no 853/2004 of the European parliament and of the council of 29 April 2004 laying down specific hygiene rules for food of animal origin foodstuffs. Off J Eur Union 139:151

9. Hoogenboom L (Ron), Knutsen HK, Rose M, Grasl‐Kraupp B, Wallace H, Viviani B, Hogstrand C, Horvath Z, Binaglia M, Barregård L, Alexander J, Petersen A, Arcella D, Arnich N, Edler L, Schwerdtle T, Benford D, Cottrill B, Ceccatelli S, Dinovi M, Vleminckx C, Oswald IP, Roudot A, van Manen M, Steinkellner H, Brüschweiler B, Nebbia CS, Botana L, Vollmer G, Bignami M (2017) Risks for public health related to the presence of tetrodotoxin (TTX) and TTX analogues in marine bivalves and gastropods. EFSA J 15: . doi: 10.2903/j.efsa.2017.4752

10. Chand P (2009) CHAPTER 40 - Seafood Neurotoxins I: Shellfish Poisoning and the Nervous System. In: DOBBS MRBT-CN (ed). W.B. Saunders, Philadelphia, pp 441–447

11. Mcvey C, Logan N, Thanh NTK, Elliott C, Cao C (2019) Unusual switchable peroxidase-mimicking nanozyme for the deter- mination of proteolytic biomarker. Nano Res 12:1–8 . doi: 10.1007/s12274-018-2241-3

12. Wang J, Pedrero M, Sakslund H, Hammerich O, Pingarron J (1996) Electrochemical Activation of Screen-printed Carbon Strips. Analyst 121:345–350
